# Intrahepatic Cholestasis of Pregnancy (ICP) in U.S. Latinas and Chileans: Clinical features, Ancestry Analysis, and Admixture Mapping

**DOI:** 10.1371/journal.pone.0131211

**Published:** 2015-06-30

**Authors:** Laura N. Bull, Donglei Hu, Sohela Shah, Luisa Temple, Karla Silva, Scott Huntsman, Jennifer Melgar, Mary T. Geiser, Ukina Sanford, Juan A. Ortiz, Richard H. Lee, Juan P. Kusanovic, Elad Ziv, Juan E. Vargas

**Affiliations:** 1 Liver Center Laboratory, Department of Medicine, University of California San Francisco, San Francisco, California, United States of America; 2 Institute for Human Genetics, University of California San Francisco, San Francisco California, United States of America; 3 Division of General Internal Medicine, Department of Medicine, University of California San Francisco, San Francisco, California, United States of America; 4 Center for Research and Innovation in Maternal-Fetal Medicine, Hospital Dr. Sótero del Río, Puente Alto, Chile; 5 Department of Obstetrics and Gynecology, Hospital Dr. Sótero del Río, Puente Alto, Chile; 6 Department of Obstetrics and Gynecology, Clínica Santa María, Santiago, Chile; 7 Division of Maternal-Fetal Medicine, Department of Obstetrics and Gynecology, Keck School of Medicine, Los Angeles County + University of Southern California Medical Center, Los Angeles, California, United States of America; 8 Division of Obstetrics and Gynecology, School of Medicine, Pontificia Universidad Católica de Chile, Santiago, Chile; 9 Department of Obstetrics, Gynecology and Reproductive Sciences, University of California San Francisco, San Francisco, California, United States of America; 10 Department of Radiology, University of California San Francisco, San Francisco, California, United States of America; Icahn School of Medicine at Mount Sinai, UNITED STATES

## Abstract

In the Americas, women with Indigenous American ancestry are at increased risk of intrahepatic cholestasis of pregnancy (ICP), relative to women of other ethnicities. We hypothesized that ancestry-related genetic factors contribute to this increased risk. We collected clinical and laboratory data, and performed biochemical assays on samples from U.S. Latinas and Chilean women, with and without ICP. The study sample included 198 women with ICP (90 from California, U.S., and 108 from Chile) and 174 pregnant control women (69 from California, U.S., and 105 from Chile). SNP genotyping was performed using Affymetrix arrays. We compared overall genetic ancestry between cases and controls, and used a genome-wide admixture mapping approach to screen for ICP susceptibility loci. We identified commonalities and differences in features of ICP between the 2 countries and determined that cases had a greater proportion of Indigenous American ancestry than did controls (p = 0.034). We performed admixture mapping, taking country of origin into account, and identified one locus for which Native American ancestry was associated with increased risk of ICP at a genome-wide level of significance (*P* = 3.1 x 10^-5^, *P_corrected_* = 0.035). This locus has an odds ratio of 4.48 (95% CI: 2.21-9.06) for 2 versus zero Indigenous American chromosomes. This locus lies on chromosome 2, with a 10 Mb 95% confidence interval which does not contain any previously identified hereditary ‘cholestasis genes.’ Our results indicate that genetic factors contribute to the risk of developing ICP in the Americas, and support the utility of clinical and genetic studies of ethnically mixed populations for increasing our understanding of ICP.

## Introduction

Among pregnant women, the most common pregnancy-specific hepatic disorder is intrahepatic cholestasis of pregnancy (ICP). Patients with ICP typically present in the third trimester with a characteristic pruritus pattern that involves the palms and soles in the absence of a skin rash. For the mother, ICP is usually a benign condition that promptly resolves after birth; however ICP has been associated with a higher incidence of adverse pregnancy outcomes, including preterm delivery, non-reassuring fetal status, meconium staining of the amniotic fluid, and stillbirth [1–3].

Incidence of ICP differs between populations, and in some, has changed over time, suggesting both genetic and environmental contributions to etiology [4]. In European populations, cross-sectional and candidate gene studies support a contribution of genetic factors to ICP; the candidate genes most consistently implicated in ICP are *ABCB11* and *ABCB4* [5–16]. Nevertheless, additional genetic factors contributing to ICP remain to be identified. No whole-genome studies of ICP have been reported.

Historically, reported incidence of ICP has been highest in Chile and Bolivia, especially amongst women of Indigenous American ancestry. Nevertheless, few genetic studies of ICP in women with Indigenous American ancestry have been performed, and these did not quantitatively assess nor factor ancestry into the analyses [17,18].

Recent studies of ICP in the U.S. confirm that it is more common in Latinas (incidences of 1.9% and 5.6% in predominantly Latina California obstetric populations), while it is relatively rare in obstetric populations that have a low proportion of Latinas (incidences of 0.32% and 0.25%) [19–22]. Two single-center studies of ethnically heterogeneous obstetric populations confirm that Latinas are at increased risk of ICP; in our study of such a population in San Francisco, Latinas had a substantially increased probability of having ICP (RR 4.96; 95% CI 2.59–9.51) compared to women of other ancestries [19]. In a recent study at a Rhode Island medical center, an odds ratio of 2.79 (95% CI 1.94–4.00) for risk of ICP in Latinas compared to European Americans was obtained [21].

Such results suggest that genetic factors contribute to increased risk of ICP in women with Indigenous American ancestry. The use of the statistically powerful genetic ‘admixture mapping’ (sometimes termed ‘ancestry mapping’) approach could allow mapping of susceptibility loci underlying the increased risk of ICP in such women, even in a modestly-sized sample, if a limited number of distinct genetic factors contribute meaningfully to ICP’s greater incidence in women with Indigenous American ancestry [23].

## Materials and Methods

### Human Subjects

Study protocols were approved by the IRBs of the sites involved, including University of California San Francisco (UCSF), University of Southern California (USC) and Sótero del Río Hospital (Santiago, Chile), and written documentation of consent was obtained. The subset of enrolled women identified as Latina/Hispanic were included in this study. The descriptions below apply to the Hispanic enrollees in whom genotyping for this study was successfully completed.

### U.S. ICP cases and pregnant controls

Enrolled affected and unaffected women were administered a questionnaire and blood draw for isolation of DNA and preservation of plasma (women were not asked to fast before the blood draw), and relevant clinical data was collected from available medical records. Seventy-one women diagnosed with ICP were recruited at two UCSF hospitals and affiliated clinics [most were recruited at San Francisco General Hospital (SFGH) and a few at UCSF Medical Center] and nineteen women with ICP at Los Angeles County + University of Southern California Medical Center. Seventy-three affected women were recruited and underwent sample collection on or before date of delivery, and 17 women underwent sample collection after delivery of the affected pregnancy.

Potential pregnant control women were identified and enrolled in San Francisco, through pre-screening of women with prenatal clinic appointments, and use of recruitment flyers. Most control women were receiving clinical care at SFGH, but 4 were seen at UCSF Medical Center or other nearby clinics. All 69 control women were enrolled, and underwent sample collection, on or before date of delivery.

### Chilean ICP cases and pregnant controls

Enrolled affected and unaffected women were administered a questionnaire and blood draw for isolation of DNA and preservation of serum, and relevant clinical data was collected from available medical records. All affected and unaffected women were enrolled at the Sótero del Río Hospital, affiliated with the Pontifical Catholic University of Chile. The blood draw was performed on or before the date of delivery for 106 of 108 affected women enrolled, and for all 105 control women enrolled.

### Clinical diagnosis of ICP

Sites diagnosed ICP based on their clinical experience. Diagnostic criteria included the following: 1) clinical presentation of severe itching without a rash (except for excoriation), with a pattern of distribution characteristic of ICP, which includes the hands and feet (mainly palms and soles) [24]; 2) exclusion of other etiologies for clinical and biochemical findings, such as other forms of hepatic disease; in particular, patients with high serum AST and/or ALT were only included if hepatitis A, B, and C, severe preeclampsia with liver involvement, HELLP syndrome, acute fatty liver of pregnancy, drug toxicities/exposures and/or other known causes of hepatic impairment were excluded as possible etiologies for hepatic disease. Also, disappearance of pruritus within a month of delivery was confirmed. 3) Results of serum biochemistry available from medical records, variously including serum AST, ALT, bilirubin (total and direct) and total serum bile acids (TBA), also contributed to diagnosis. Eighty-seven percent (n = 78) of U.S. cases had documented elevated TBA and/or transaminases during the study pregnancy. Twelve U.S. cases had normal and/or unavailable serum biochemistry for the study pregnancy, six of whom had a history of ICP in a previous pregnancy, and six of whom were diagnosed based upon criteria 1 and 2. At the Chilean site, TBA are not measured clinically for diagnosis of ICP, and while a subset of the women had elevated transaminases and/or bilirubin, many were diagnosed based upon clinical criteria.

### Biochemical studies of serum and plasma collected for the study

Serum (Chile) and plasma (U.S.) samples from participants were assayed for AST, ALT, albumin, total protein, and total and direct bilirubin by the clinical laboratory at SFGH; these assays have been validated for use on both plasma and serum at this laboratory. These study samples were assayed for total serum bile acid concentration, and activity of γ-glutamyl-transpeptidase, in a research laboratory, using the Total Bile Acids Assay Kit (Diazyme) and γGT reagent (Thermo Scientific). Results of these assays, in study participants from whom the study sample was collected on or prior to date of delivery, are reported.

### Selection of control pregnant women

Within the Chilean and U.S. samples, we endeavored to achieve similar overall maternal age and gestational age at study blood draw (for those women pregnant at time of blood draw) between cases and controls. Control women were having low-risk, uncomplicated pregnancies at time of enrollment, with special attention focused on confirming lack of liver disease or skin conditions. Clinical data on the completed pregnancy was collected, and samples from women who did not develop pregnancy complications involving the liver or skin (which could represent a form of ICP) after enrollment were used in this study.

### Genotyping and quality control (QC) procedures

ICP cases and pregnant controls were genotyped at the UCSF Genomics Core Facility using the Affymetrix Axiom Genome-wide LAT 1 array, which is optimized for study of populations with mixed ancestry including Indigenous American, European, and West African ancestry. QC of samples included exclusion of samples with a call rate of <97% and a check for relatedness between samples; after QC, 376 (~99%) samples yielded adequate data for inclusion. QC of SNPs was as follows: filtering using SNP heterozygous strength offset (HetSO) and Fisher’s linear discriminant analysis for SNPs was applied as recommended by Affymetrix, and SNPs with >5% missing data, or with discordance across replicates, were excluded.

### Global and Locus-specific ancestry estimation

Calculation of locus-specific and global ancestry: We first selected a subset of markers that had ancestral information in the reconstructed reference panel from Pasaniuc *et al*. [25]. Pasaniuc *et al*. [25] developed a reference dataset from 489 Mexican and Puerto Rican trios (978 independent individuals) genotyped on an Affymetrix 6.0 array. They initially calculated the locus-specific ancestry in these trios using external ancestral reference panels (Hap Map phase 3 and [26]) and determined regions at which there were Mendelian inconsistencies. They then leveraged this analysis to infer haplotype frequencies in the ancestral populations of Latino populations (Europeans, Africans and Native Americans) and created a new reference dataset for these ancestral populations. We used the reconstructed ancestral frequencies from these 978 individuals as our ancestry reference panel, resulting in a reference panel larger than our total study sample. Of the 664,016 markers on the Axiom LAT 1 array that we used, 109,917 were also contained in the reconstructed reference panel, and so could be included in the ancestry estimation. Although we therefore did not use most of the markers on the Axiom array, we reasoned that it would be better to work with a smaller set of markers with better ancestral haplotype frequencies, than to work with more markers and use less robust estimates of ancestral haplotype frequencies, since larger reference panels are key to reducing error in locus-specific ancestry estimation [25]. We set LAMP-LD to the default settings, which included a window length of 300 SNPs and 15 states. Then, we used LAMP-LD [27,28] to estimate locus-specific ancestry at the position of each SNP in the dataset. LAMP-LD performs ancestry estimation using a hidden Markov model of haplotype diversity within a windows based approach. Global ancestry for each individual was estimated as the average locus-specific ancestry across 109,917 loci using LAMP-LD [27]. Genome-wide ancestry was calculated, excluding the X-chromosome.

### Association analysis

Comparing ancestry in cases and controls: To compare the mean ancestry of cases and controls, we used a permutation procedure. First, we calculated the T-statistic for comparison of means. Then we calculated the empirical distribution of the null hypothesis by permuting case-control status and re-calculating the T-statistic 10,000 times. In each permutation, we permuted the Chilean and U.S. samples as two separate strata. We compared the rank of absolute value of the T-statistic from the observed data to the absolute values of the T-statistics from the distribution from permutations to determine the p-value.

### Admixture mapping

To perform association analysis for locus-specific ancestry (admixture mapping) we used logistic regression models, where the outcome was case vs. control. The predictor variables included the ancestry at each SNP for each individual as a continuous variable. Adjustments were made for country of enrollment and for overall genetic ancestry of each individual. (Percentage Indigenous American and percentage African were both entered into each model. Percentage European ancestry is accounted for automatically by entering the other 2 ancestry components, since it is completely determined by them.) We ran three models for each SNP of the 109,917 SNPs, entering the locus-specific ancestry as either Indigenous American, European or African. However, since our prior for Indigenous American ancestry as a risk factor was highest and since African ancestry was low in the sample, we focused on the models that tested Indigenous American locus-specific ancestry.

Genome-wide significance for admixture mapping depends on the effective number of independent loci tested. Since admixture linkage disequilibrium extends over distances of megabases in Latin American populations, the number of independent loci tested is far fewer than 100,000. We empirically calculated the threshold for genome-wide significance using a permutation-based approach. Cases and controls were permuted using 4 bins of ancestry (0–0.25, 0.25–0.5, 0.5–0.75, 0.75–1) and for each permutation we repeated the genome-wide locus-specific ancestry association analysis, adjusted for overall ancestry and recorded the lowest p-value [29]. We ran 1000 permutations and compared the distribution of lowest p-values from these permutations with the lowest p-value from the case-control analysis.

### Other statistical analyses

Analysis of participant characteristics, clinical and laboratory data was performed using GraphPad Prism (GraphPad Software, La Jolla California, USA) and nominal p-values are presented. For categorical variables, Fisher’s exact test was performed. When data for continuous variables were not normally distributed, they were transformed. On normally distributed data, the t-test was performed, with Welch’s correction for differing variances, where appropriate. For variables where distribution was not normalized by transformation, the Mann-Whitney test (MW) on nontransformed data was used instead.

## Results

### Participant and sampling characteristics (Table 1)

A total of 198 women with ICP and 174 pregnant controls were included in this study. Chilean participants were younger than U.S. participants (p<0.0001, MW), but ages of cases and controls within the U.S. and Chilean samples were similar. Reported week of ICP onset during pregnancy was modestly later in the Chilean, than U.S., patients (p = 0.0042, MW). Number of previous births was assessed, and was similar between the U.S. cases and controls, although in the Chilean sample, women with ICP had more prior births than did the controls. Most women did not report any prior spontaneous miscarriages or stillbirths, and no difference between cases and controls within a country was detected. This study included only two twin pregnancies—one case each amongst U.S. and Chilean women with ICP.

**Table 1:** Participant and sampling characteristics.

Characteristic Median (IQR), N	US Cases (N = 89^)	US Controls (N = 69)	P-value (cases vs controls)	Chilean Cases (N = 108)	Chilean Controls (N = 105)	P-value (cases vs controls)
Age* (years)	28 (25–33) N = 89	28 (25–31) N = 69	0.2154	23 (20–30) N = 108	22 (20–27) N = 105	0.2731
Gestational age (weeks) at reported onset of symptoms	31 (28–34) N = 75	Not applicable		33 (30–35) N = 104	Not applicable	
# previous births	1 (0–2) N = 86	1 (0–2) N = 69	0.5746	1 (0–2) N = 107	0(0–1) N = 105	**0.0187**
Gestational age at study blood draw	36 (34–37) N = 69	36 (33–38) N = 69	0.6619	38 (37–39) N = 105	39 (39–40) N = 105	**<0.0001**

*Age at blood draw, or for retrospectively enrolled women, reported age during the ICP-affected study pregnancy. ^Data for 89 of 90 US cases is included, as due to sample labelling uncertainty, one sample could have come from either of 2 enrolled affected women, so data from that sample is excluded here. Maternal age was analyzed using the t-test, and other variables, using the Mann-Whitney test.

For those women who had their study blood samples drawn before or on the date of delivery, gestational age at study blood draw was well-matched between cases and controls in the U.S. sample. In the Chilean sample, the median gestational age of cases differed from that of controls, but was only one week greater in controls. Overall, U.S. participants had their samples drawn a median of 2–3 weeks earlier in pregnancy than did the Chilean participants.

### Ethnicity and country of origin of participants (Table 2)

All women included in the U.S. and Chilean ICP and pregnant control samples self-identified as Latina or Hispanic. The women in the U.S. sample had a diverse range of countries of birth in the Americas, with Mexico as the most frequently reported. Also, most U.S. participants born in the U.S. had parents or grandparents born outside the U.S. All Chilean participants were born in Chile, except for 1 case born in Venezuela, and 1 control born in Argentina (but whose grandparents were born in Chile). Of the women born in Chile, only 2 (1 case, 1 control) had a single grandparent born outside Chile.

**Table 2:** Countries of Birth for U.S. Participants.

Country	US Cases # (%) (N = 88)	US Controls # (%) (N = 69)
Chile	0 (0%)	1 (1.5%)
El Salvador	9 (10.2%)	11 (15.9%)
Guatemala	11 (12.5%)	7 (10.1%)
Honduras	1 (1.1%)	5 (7.2%)
Mexico	51 (58.0%)	25 (36.2%)
Nicaragua	2 (2.3%)	4 (5.8%)
Peru	1 (1.1%)	2 (2.9%)
U.S.	13 (14.8%)	14 (20.3%)

### Laboratory findings (Tables 3 and 4)

As this is not a clinical trial, we collected information from medical records, and did not request that study sites alter clinical practices to accommodate the study. Therefore, as expected, there are differences between sites and patients in management and diagnostic testing. We collected results of clinical laboratory testing for prospectively and retrospectively enrolled case and control women from all sites, and present analysis of the highest (or only) laboratory value for each parameter tested (Tables 3 and 4, ‘Clinical’). To complement available clinical data, we performed biochemical assays on study samples collected before or on the date of delivery (Tables 3 and 4, ‘Research’). Table 3 includes median and interquartile ranges for these data; for those biochemical variables for which we could define a cutoff between normal and abnormal, Table 4 reports the proportion of assayed patients with abnormal values. Results for total protein and albumin are shown in S1 and S2 Tables.

**Table 3:** Median laboratory findings.

Laboratory test	US Cases Median (IQR)	US Controls Median (IQR)	p-value (cases vs controls)	Chilean Cases Median (IQR)	Chilean controls Median (IQR)	p-value (cases vs controls)
TBA (umol/L) (Clinical)	15.5 (11.7–38.7) (N = 84)	—		—	—	
TBA (umol/L) (Research)	20 (10–38) (N = 71)	4 (3–8) (N = 69)	**<0.0001**	6 (2–17) (N = 106)	2 (0–4) (N = 105)	**<0.0001**
Total Bilirubin (mg/dL) (Clinical)	0.5 (0.4–0.7) (N = 72)	—		0.4 (0.3–0.7) (N = 58)	—	
Total Bilirubin (mg/dL) (Research)	0.5 (0.3–0.6) (N = 72)	0.3 (0.3–0.4) (N = 69)	**<0.0001**	0.4 (0.3–0.5) (N = 106)	0.4 (0.3–0.5) (N = 105)	0.9193
Direct Bilirubin (mg/dL) (Clinical)	0.15 (0.1–0.3) (N = 62)	—		1.08 (0.63–1.84) (N = 9)	—	
Direct Bilirubin (mg/dL) (Research)	0.30 (0.20–0.30) (N = 72)	0.20 (0.20–0.20) (N = 69)	**<0.0001**	0.20 (0.20–0.30) (N = 106)	0.20 (0.20–0.25) (N = 105)	0.2785
ALT (U/L) (Clinical)	61 (28–153) (N = 89)	—		19 (12–70) (N = 62)	—	
ALT (U/L) (Research)	46 (22–119) (N = 72)	17 (13–20) (N = 69)	**<0.0001**	14 (10–27) (N = 106)	12 (10–15) (N = 105)	**0.0030**
AST (U/L) (Clinical)	43 (31–87) (N = 89)	—		23 (15–45) (N = 63)	—	
AST (U/L) (Research)	43 (32–65) (N = 72)	23 (19–30) (N = 69)	**<0.0001**	27 (20–36) (N = 106)	26 (22–31) (N = 105)	0.7520
γGT (U/L) (Clinical)	16 (14–17) (N = 3)	—		17 (11–27) (N = 44)	—	
γGT (U/L) (Research)	11 (4–31) (N = 71)	4 (0–11) (N = 69)	**0.0008**	4 (0–11) (N = 106)	2 (0–11) (N = 105)	0.2581

Table 3 notes. For some biochemical parameters, results from medical records were available from a subset of controls; those data are not presented, as there is likely bias in selection of which control women would undergo testing for clinical purposes. Therefore, comparisons between cases and controls are performed only for data from the study samples. P-values are from the Mann-Whitney test.

**Table 4:** Normal versus abnormal laboratory findings.

Laboratory test, with cutoff for normal range	US Cases % abnormal	US Controls % abnormal	p-value (cases vs controls)	Chilean Cases % abnormal	Chilean controls % abnormal	p-value (cases vs controls)
TBA (Clinical) (< 10 umol/L)	81% elevated	—		—	—	
TBIL (Clinical) (≤ 1.0 mg/dL)	13% elevated	—		14%	—	
TBIL (Research) (≤ 1.0 mg/dL)	3% elevated	0%	0.4967	3%	0%	0.2464
DBIL (Clinical) (≤ 0.3 mg/dL)	18% elevated	—		(89%)	—	
DBIL (Research) (≤ 0.3 mg/dL)	22% elevated	1%	**<0.0001**	18%	6%	**0.0095**
ALT (Clinical) (≤ 35 U/L)	67% elevated	—		34%	—	
ALT (Research) (≤ 35 U/L)	60% elevated	3%	**<0.0001**	21%	3%	**<0.0001**
AST (Clinical) (≤ 41 U/L)	53% elevated	—		29%	—	
AST (Research) (≤ 41 U/L)	53% elevated	7%	**<0.0001**	22%	4%	**<0.0001**
gGT (Clinical) (5–36 U/L)	(0%)	—		16%	—	

Table 4 notes. For some biochemical parameters, results from medical records were available from a subset of controls; those data are not presented, as there is likely bias in selection of which control women would undergo testing for clinical purposes. Therefore, comparisons between cases and controls are performed only for data from the study samples. Normal range definitions: Since study samples were assayed for a number of parameters at the SFGH clinical laboratory, we used normal ranges in use at SFGH for the analyses presented in Table 4, except for γGT, for which the normal range at the Chilean site is indicated, as that is the only site where appreciable numbers of samples were assayed for γGT in a clinical laboratory. The normal ranges used are the standards for nonpregnant women; as designation and application of pregnancy-specific ranges for these tests is inconsistent between laboratories, we did not use them. To note, for results of biochemical tests from medical records, normal ranges sometimes differ modestly between study sites; while using such site-specific normal ranges for reporting of the ‘clinical’ data in Table 4 could change percentages somewhat, the general trends remain, so the same ‘consensus’ ranges were used for data from all sites, to avoid complexities in presentation. For ‘research’ TBA and γGT, normal ranges are not indicated, and results are only presented in Table 3, as the tests performed were not clinical laboratory assays with well-defined normal ranges. Percentages in parentheses in Table 4 are of questionable meaning, due to small sample size. P-values are from Fisher’s exact test.

Reported use of medications to treat ICP is more common in the U.S. than Chilean cases; prescription of ursodeoxycholic acid (UDCA) was noted for 48.3% (43/89) of U.S. cases, but for only 1 Chilean case. This difference in medication use may influence results of some laboratory tests, in cases where samples were collected while a woman was taking medication.

### Total bile acids (TBA)

From medical records, total bile acid results were available from most U.S. cases, but not from other study samples. A commonly used cutoff for considering TBA elevated in ICP is ≥10 umol/L; by this standard, 81% of U.S. ICP cases had elevated clinically-assayed TBA (Tables 3 and 4). TBA were also assayed on study samples in a research laboratory; results are likely not directly comparable to clinically assayed TBA, but appropriate case and control datasets can be compared. Within each country, TBA of cases were elevated compared to controls (Table 3). The overlap between cases and controls was greater in the Chilean sample, consistent with the fact that TBA are not used in clinical diagnosis of ICP at the Chilean study site. There was a systematic difference in results between countries, with U.S. cases and controls having higher median values than did the corresponding Chilean samples (P<0.0001 for both comparisons, MW; Table 3). The greater elevation in U.S. than Chilean cases can likely be partially attributed to the use of TBA as a diagnostic test for ICP in the U.S., but this does not explain the difference in controls between countries.

### Bilirubin

Total bilirubin (TBIL) from medical records was elevated (>1.0 mg/dL) in 13% of U.S. and 14% of Chilean cases tested, respectively (Table 4). TBIL assayed on the research samples was modestly higher in U.S. cases than controls, but similar between Chilean cases and controls (Table 3); 3% each of U.S. and Chilean cases had values >1.0 mg/dL, while no controls did (Table 4).

Direct bilirubin (DBIL) from medical records was available from 62 U.S. cases, of whom 18% had elevated levels; eight of the nine Chilean cases assayed had elevated DBIL, likely due to greater probability of performing the assay in women who appeared more severely affected (Table 4). As with TBIL, distribution of DBIL assayed on study samples was modestly higher in U.S. cases than controls, while Chilean cases and controls appeared similar (Table 3). However, for both U.S. and Chilean samples, a greater proportion of patients had elevated levels than did controls (Table 4).

### Transaminases

Alanine aminotransferase (ALT) and aspartate aminotransferase (AST) are higher in U.S. cases than U.S. controls. In the Chilean study samples, distribution of ALT, but not AST, trends higher in cases than controls (Table 3). When study sample results are dichotomized into normal or elevated (for ALT, >35 U/L; for AST, >41 U/L), the proportion of cases with elevated ALT or AST is substantially higher compared to controls for both U.S. and Chilean samples (Table 4).

### Gamma-glutamyltranspeptidase (γGT)

Since this biochemical parameter is not routinely measured in ICP at our U.S. study sites, and only in a portion of Chilean patients (16% of whom had an elevated value [Table 4]), we assayed it on the study samples in a research lab; similar to the result with TBA, these results may not be directly comparable with clinical testing, but cases and controls can be compared; γGT was significantly higher in cases than controls in the U.S. sample, but not significantly different in the Chilean sample (Table 3).

### Pregnancy and neonatal outcomes (Table 5)

Most women underwent normal spontaneous vaginal birth, but a greater proportion of Chilean cases were delivered by cesarean birth, and fewer by spontaneous vaginal birth, than was the case in the other 3 groups. Median birthweights (excluding twins) were similar between Chilean cases and controls, but babies born to U.S. cases were smaller than those born to U.S. controls. This is likely because most U.S. cases were delivered by 37 weeks, or at the time of diagnosis if this occurred beyond 37 weeks, to avoid the highest-risk period for poor fetal outcome at the end of pregnancy, so their gestational ages were modestly lower than the controls; induction of labor due to ICP is not performed until 40 weeks gestation at the Chilean site, in the absence of hyperbilirubinemia or other pregnancy complications. Excluding twins, median 1-minute Apgar score was 8 or 9 in all groups, and median 5-minute Apgar score was 9 in all groups. One neonatal death occurred in a Chilean ICP case, attributed to placental abruption.

**Table 5:** Pregnancy and neonatal outcomes.

Characteristic	US Cases (N = 90)	US Controls (N = 69)	P-value (cases vs controls)	Chilean Cases (N = 108)	Chilean Controls (N = 105)	P-value (cases vs controls)
Normal Spontaneous Vaginal Birth	65 (74%) (N = 88)	50 (77%) (N = 65)	0.7084	61 (59%) (N = 104)	79 (75%)	**0.0125**
Assisted Vaginal Birth (vacuum, forceps)	2 (2%) (N = 88)	2 (3%) (N = 65)		3 (3%) (N = 104)	1 (1%)	
Cesarean Birth	21 (24%) (N = 88)	13 (20%) (N = 65)	0.6946	40 (38%) (N = 104)	25 (24%)	**0.0254**
Birth weight* (g; median and IQR)	3125 (2733–3370) (N = 80)	3477 (3123–3785) (N = 50)	**<0.0001**	3425 (3090–3730) (N = 103)	3410 (3075–3725) (N = 105)	0.7196
Gestational age at birth (weeks; median and IQR)	37 (36–38) (N = 83)	40 (39–41) (N = 65)	**<0.0001**	40 (38–40) (N = 101)	40 (39–40) (N = 104)	0.6810

Notes: ‘*’ = Excluding twins. Vaginal and Cesarean birth were analyzed using Fisher’s exact test, birth weight using the t-test, and gestational age using the Mann-Whitney test. Where ‘N’ for a cell is lower than the total N at the top of the column, N for that cell is indicated.

### Genetic ancestry in cases and controls (Table 6)

We compared the overall genetic ancestry in cases versus controls. In the complete dataset, cases had higher Indigenous American ancestry compared to controls (Table 6). When analyzed separately by country, U.S. cases had greater Indigenous American ancestry than controls, while Chilean cases and controls were similar (Table 6). Power to detect an association with genetic ancestry is driven by the variation in individual ancestry in a sample. The U.S. study sample had a much higher standard deviation of ancestry (Table 6). Overall, we found an odds ratio of 3.91 (95% CI 1.1–13.7) for Indigenous American ancestry as a risk factor (on a scale of 0–100% Indigenous American ancestry). Our analysis is consistent with the epidemiological observation that populations with Indigenous American ancestry have higher prevalence of ICP than do those of European ancestry without Indigenous American ancestry.

**Table 6:** Genome-wide Ancestry in Cases and Controls.

Sample	Mean European Ancestry (SD)	Mean Indigenous American Ancestry (SD)	Mean African Ancestry (SD)	P-value for cases vs controls, Indigenous American Ancestry
Total ICP cases (N = 198)	44.0% (17.4%)	53.0% (17.6%)	3.0% (3.3%)	**0.034**
Total pregnant controls (N = 174)	47.4% (15.6%)	49.3% (15.6%)	3.3% (6.8%)	
US ICP cases (N = 90)	31.4% (17.5%)	64.2% (19.4%)	4.4% (4.4%)	**0.034**
US pregnant controls (N = 69)	36.6% (18.2%)	57.4% (20.4%)	6.0% (10.2%)	
Chilean ICP cases (N = 108)	54.6% (7.8%)	43.7% (8.0%)	1.8% (1.0%)	0.84
Chilean pregnant controls (N = 105)	54.5% (7.8%)	43.9% (7.9%)	1.5% (0.7%)	

Note: these results exclude the X chromosome. P-values were calculated using a permutation procedure, to avoid assumptions about normality.

### Genome-Wide Admixture mapping for ICP

**Fig 1:**
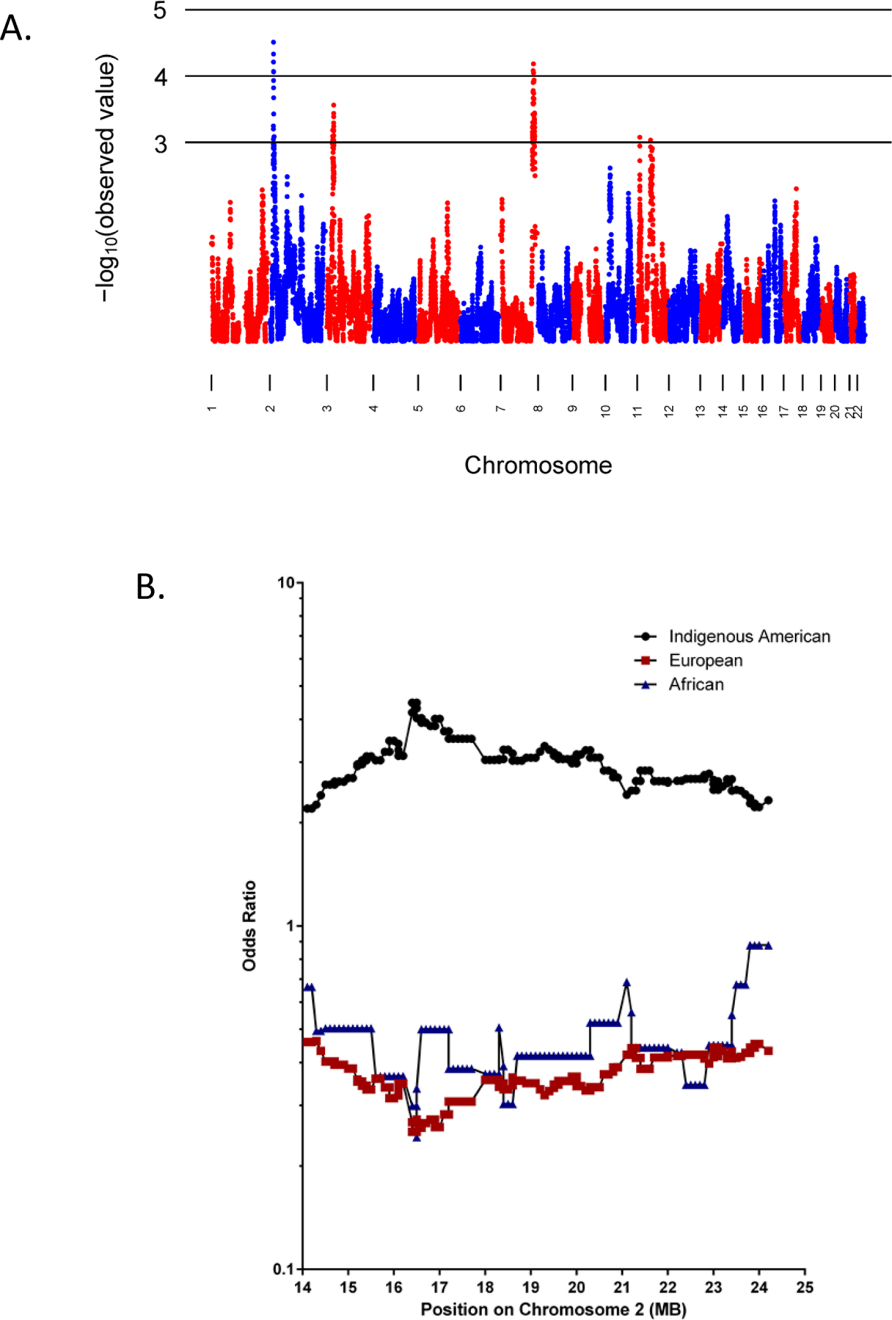
Results of admixture mapping for the Indigenous American component. Fig 1A. X-axis: positions by chromosome. Y-axis: negative log_10_ P-values for the association between ICP and locus-specific ancestry. Fig 1B. X-axis: genomic position in the chromosome 2 locus. Y-axis: Fold-increased or–decreased odds of ICP attributed to 1) risk associated with Indigenous American ancestry (black); 2) risk associated with European ancestry (red); and 3) risk associated with African ancestry (blue). The locus-specific ancestry is coded such that the OR is for 2 chromosomes from the population being tested (Indigenous American, European, or African) versus zero chromosomes from that population.

We performed genome-wide admixture mapping for ICP (Fig 1, S1 Fig). Indigenous American ancestry was associated with increased risk of ICP at p-values of < 10^−4^ at regions on chromosomes 2 and 7 (Fig 1, Table 7). We used a permutation procedure to assess the evidence for genome-wide significance. We found an empirical genome-wide p-value that surpassed the threshold for genome-wide significance for the chromosome 2 locus, but not for the locus on chromosome 7 (Table 7). Of note, at both of these loci, Indigenous American ancestry was associated with higher risk of ICP, as reflected in the odds ratios (Table 7), and consistent with the epidemiology.

**Table 7:** Loci with strongest P-values from admixture mapping of the Indigenous American component.

Chromosome	Odds ratio* (95% CI)	P-value	Genome-wide P-value	Chromosomal position of SNP with lowest P-value (bp)	95% CI of chromosomal position (bp) (flanking SNPs)
2	4.48 (2.21–9.06)	3.1 x 10^−5^	**0.035**	16,419,276	14,116,991 to 24,247,686 (rs10495620 to rs7600852)
7	4.19 (2.07–8.47)	6.5 x 10^−5^	0.08	142,922,662	137,890,026 to 150,455,295 (rs11761248 to rs10266177)

*Fold-increased odds of ICP attributed to homozygosity for Indigenous American alleles at the locus, relative to zero Indigenous American alleles.

Note: genomic positions are derived from version GRCh37/hg19 of the human genome sequence.

The 95% confidence interval (CI) for the chromosome 2 locus is ~10 Mb in size (Table 7), and does not contain any genes previously identified as associated with hereditary cholestasis; a list of the Refseq genes in the 95% CI is shown in S3 Table. Separate analysis of the U.S. and Chilean study samples at the locus on chromosome 2 indicates that data from both samples contribute to the positive finding.

## Discussion

This study represents the first analysis of genetic ancestry and ICP among admixed populations in the Americas and the first genome-wide admixture mapping for this trait. We demonstrated a significant association between higher Indigenous American ancestry and ICP. This finding is consistent with the higher ICP risk found in Hispanics compared to other populations, though the latter finding is potentially confounded by other non-genetic factors that vary between Hispanic populations and other populations. Our study did not detect an association between higher Indigenous American ancestry and ICP among the Chileans. However, the association between individual ancestry and disease phenotype depends on variation among individuals. The Chilean samples had much lower variation in individual ancestry than did the U.S. samples. Since variation in individual ancestry attenuates quickly with random mating in a population several generations after admixture, it is likely that the Chilean sample represents a population that is closer to randomly mating compared to the U.S. Hispanic population. Unlike the overall individual ancestry association which is dependent on inter-individual ancestry, admixture mapping uses the variation at locus-specific ancestry, which remains variable for many generations after admixture.

Our admixture mapping analysis identified one genome-wide significant locus at which higher Indigenous American ancestry is associated with ICP. Admixture mapping is well-suited to identify loci for traits that have large differences in prevalence among different ancestral populations. The candidate region on chromosome 2 contains approximately 40 genes, none of which have previously been implicated in hereditary cholestasis. *RDH14*, retinol dehydrogenase 14, is one promising candidate gene in the region, given the importance of retinoic acid to liver health, including attenuation of liver injury in a rodent model of cholestasis [30,31]; however, it may be premature to focus too much attention on a single candidate gene within this large region. Follow-up genetic studies of admixed populations should be performed to seek confirmation and refinement of this initial mapping, as well as to allow mapping of additional loci, when the necessary study samples and genomic data become available. Our initial results indicate that continuing such an approach will likely be productive.

In addition to yielding genetic mapping results, our study allowed comparison of currently used approaches to diagnosis and management, and of resulting outcomes, of ICP in two populations in which it is relatively common; approaches to clinical diagnosis of ICP are not standardized. We hypothesize that the higher cesarean birth rate observed among cases compared to controls at the Chilean site, but not at the U.S. sites, may be due to patients with ICP at the Chilean site being more likely to have continuous electronic fetal monitoring during labor than controls, and therefore being at a higher risk of cesarean delivery for non-reassuring fetal status. Electronic fetal monitoring during labor is not routine in Chile among uncomplicated pregnancies, and it has been consistently associated with a higher incidence of cesarean births compared to intermittent auscultation of the fetal heart tones [32]. Another explanation may be that there are higher rates of non-reassuring fetal status seen among ICP patients who were expectantly managed up to 40 weeks, as is the norm at the Chilean site, rather than 37 weeks as at the participating U.S. sites. The longer course of the disease process could account for a higher incidence of observed fetal complications during labor requiring expedited delivery by cesarean birth.

All women diagnosed with ICP in our study share the clinical feature of a pattern of pruritus characteristic of ICP. However, the amount of biochemical testing used for diagnosis, and the weight put upon it for diagnosis, differ between the U.S. and Chilean sites. Therefore, it is not surprising that ICP appears overall somewhat more severe biochemically in the US, compared to the Chilean, study sample. Some of these biochemical differences seen between the U.S. and Chilean participants may also relate to differences in age, medication usage, and possible differences in BMI (unavailable), or in incidence of underlying nonalcoholic fatty liver disease or diabetes, between the study populations, or to differences in sample types (serum versus plasma) or handling. Our genetic results argue, however, that at least some genetic factors are shared in common between our 2 samples of women with ICP, despite some overall differences in laboratory and clinical findings. It could be possible, for example, that some genetic factors enhance susceptibility of women to the pruritus characteristic of ICP, but that these factors may not fully overlap with those predisposing to elevated serum bile acids during pregnancy. Continued study should allow discernment of which clinical, genetic, and biochemical features best define ICP, and of whether genetic subtypes of ICP exist, differing in their features and outcomes.

## Supporting Information

S1 Fig Results of Admixture Mapping. X-axis: positions by chromosome. Y-axis: negative log_10_ P-values for the association between ICP and locus-specific ancestry. A: admixture mapping for the European component. B: admixture mapping for the African component. Note the difference in scale on the Y-axis between the figures.
(PDF)

S1 Table Median Total Protein and Albumin. (DOCX)

S2 Table Normal versus Abnormal Total Protein and Albumin. (DOCX)

S3 Table Refseq Genes in the Chromosome 2 Candidate Region. (DOCX)
