# Correction: Intrahepatic Cholestasis of Pregnancy (ICP) in U.S. Latinas and Chileans: Clinical features, Ancestry Analysis, and Admixture Mapping

**DOI:** 10.1371/journal.pone.0137479

**Published:** 2015-08-28

**Authors:** The PLOS ONE Staff

S1 Data is missing from the list of Supporting Information. It can be viewed below. The publisher apologizes for the error.

## Supporting Information

S1 Data Genome-wide admixture mapping results for ICP. The association of ICP with locus-specific Indigenous American ancestry was tested with logistic regression. S1 Data shows the admixture mapping results for all 109917 markers across the genome.
(ZIP)
