# Supplementary figures and images for: Intrahepatic Cholestasis of Pregnancy (ICP) in U.S. Latinas and Chileans: Clinical features, Ancestry Analysis, and Admixture Mapping
Source: PLoS One. 2015 Jun 30;10(6):e0131211. doi: 10.1371/journal.pone.0131211 (PMC4488338; doi:10.1371/journal.pone.0131211)

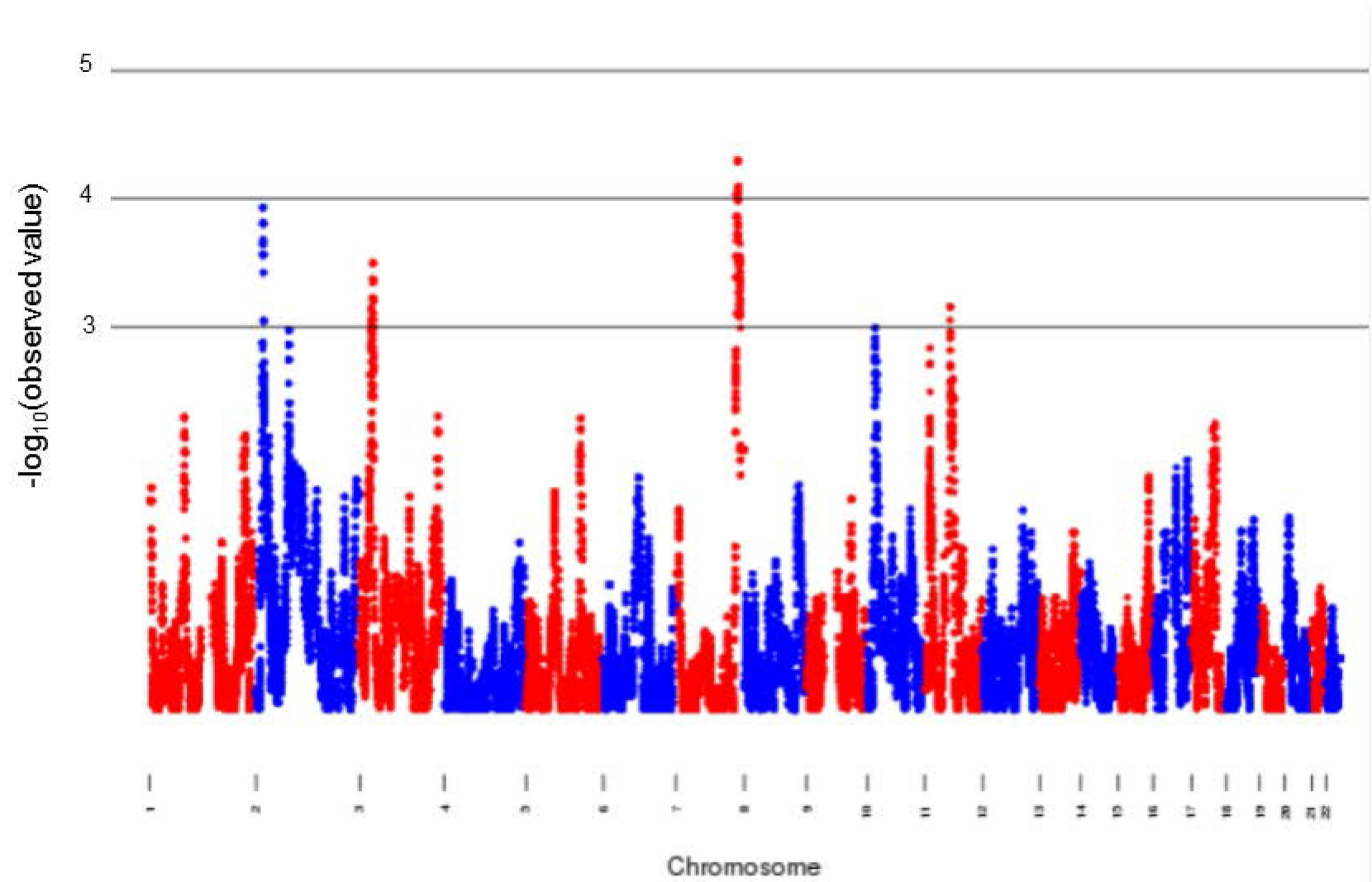

Supplementary Figure 1A.

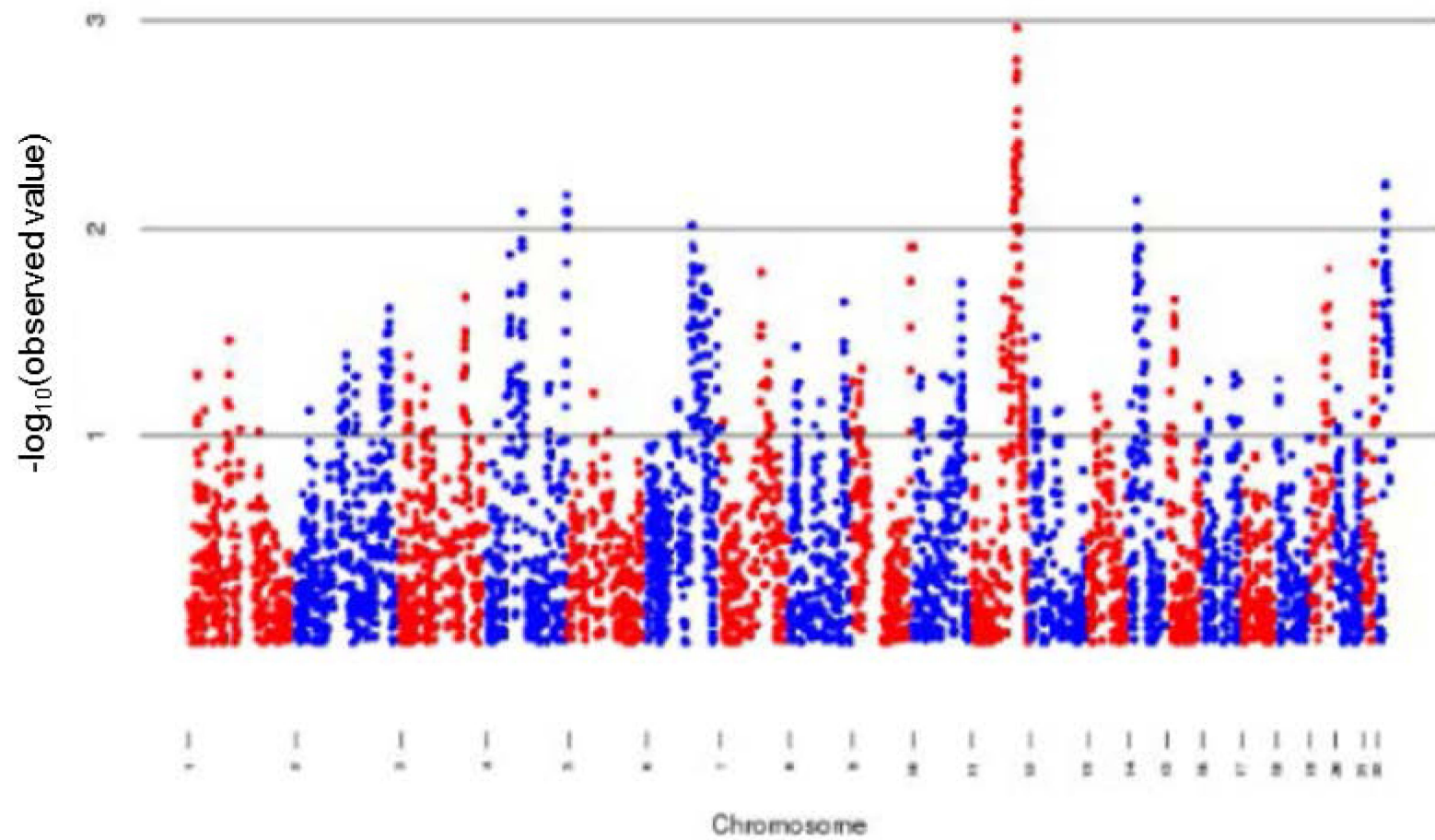

Supplementary Figure 1B.

Supplement: S1 Fig — X-axis: positions by chromosome. Y-axis: negative log10 P-values for the association between ICP and locus-specific ancestry. A: admixture mapping for the European component. B: admixture mapping for the African component. Note the difference in scale on the Y-axis between the figures. (PDF) [file pone.0131211.s001.pdf]
