# Supplementary material for: Intrahepatic Cholestasis of Pregnancy (ICP) in U.S. Latinas and Chileans: Clinical features, Ancestry Analysis, and Admixture Mapping
Source: PLoS One. 2015 Jun 30;10(6):e0131211. doi: 10.1371/journal.pone.0131211 (PMC4488338; doi:10.1371/journal.pone.0131211)
Supplement: S1 Table — (DOCX) [file pone.0131211.s002.docx]

**Supplementary Table 1. Median total protein and albumin.**

| Laboratory test | US Cases  Median (IQR) | US Controls  Median (IQR) | p-value  (cases vs controls) | Chilean Cases  Median (IQR) | Chilean controls  Median (IQR) | p-value  (cases vs controls) |
| --- | --- | --- | --- | --- | --- | --- |
| TP  (Research) | 6.8  (6.5-7.0)  (N=72) | 6.7  (6.4-6.9)  (N=69) | 0.3689 | 6.6  (6.4-6.9)  (N=106) | 6.8  (6.6-7.0)  (N=105) | **0.0193** |
| ALB  (Research) | 3.7  (3.5-3.8)  (N=72) | 3.7  (3.5-3.8)  (N=69) | 0.2250 | 3.6  (3.5-3.8)  (N=106) | 3.7  (3.6-3.9)  (N=105) | **0.0006** |
