# Supplementary material for: Intrahepatic Cholestasis of Pregnancy (ICP) in U.S. Latinas and Chileans: Clinical features, Ancestry Analysis, and Admixture Mapping
Source: PLoS One. 2015 Jun 30;10(6):e0131211. doi: 10.1371/journal.pone.0131211 (PMC4488338; doi:10.1371/journal.pone.0131211)
Supplement: S2 Table — (DOCX) [file pone.0131211.s003.docx]

**Supplementary Table 2. Normal versus abnormal total protein and albumin**

| Laboratory test, with cutoff for normal range | US Cases  % abnormal | US Controls  % abnormal | p-value  (cases vs controls) | Chilean Cases  % abnormal | Chilean controls  % abnormal | p-value  (cases vs controls) |
| --- | --- | --- | --- | --- | --- | --- |
| TP (Research)  (6.4-8.3) | 18% low | 20% | 0.8315 | 23% | 12% | 0.0694 |
| ALB (Research)  (3.4-4.8) | 14% low | 3% | **0.0313** | 8% | 2% | 0.1010 |

**Supplementary Tables 1 and 2. Total protein (TP) and albumin (ALB)** assayed on study samples. In the Chilean sample, the distribution of TP and ALB was lower in cases than controls, but this trend was not apparent in the U.S. sample (Supplementary Table 1). When results were dichotomized into normal or low, U.S. cases were more likely to have low values of ALB than were controls (Supplementary Table 2). Analyses were performed using the t-test (Supplementary Table 1) and Fisher’s exact test (Supplementary Table 2).
