# Supplementary material for: Intrahepatic Cholestasis of Pregnancy (ICP) in U.S. Latinas and Chileans: Clinical features, Ancestry Analysis, and Admixture Mapping
Source: PLoS One. 2015 Jun 30;10(6):e0131211. doi: 10.1371/journal.pone.0131211 (PMC4488338; doi:10.1371/journal.pone.0131211)
Supplement: S3 Table — (DOCX) [file pone.0131211.s004.docx]

**Supplementary Table 3.** Refseq Genes in the chromosome 2 candidate region.

| **Gene symbol** | Gene Name or classification |
| --- | --- |
| LINC00276 | Long non-coding RNA |
| FAM84A* | mRNA |
| LOC653602 | mRNA |
| NBAS | Neuroblastoma amplified sequence |
| DDX1 | DEAD box helicase 1 |
| LOC101926966 | Long non-coding RNA |
| MYCNUT | MYCN upstream transcript, long non-coding RNA |
| MYCNOS | MYCN opposite strand transcript, long non-coding RNA |
| MYCN | v-myc avian myelocytomatosis viral oncogene neuroblastoma derived homolog, mRNA |
| GACAT3 | Gastric cancer associated transcript 3 (long noncoding RNA) |
| FAM49A | mRNA |
| RAD51AP2 | RAD (recombinase) 51-associated protein 2 |
| VSNL1 | Visinin-like 1 |
| SMC6 | Structural maintenance of chromosomes 6 |
| GEN1 | Holliday junction 5’ flam endonuclease |
| MSGN1 | Mesogenin 1 |
| KCNS3 | Potassium voltage-gated channel, delayed rectifier, Subfamily S member 3 |
| NT5C1B-RDH14 | A readthrough transcript (mRNA) |
| RDH14 | Retinol dehydrogenase 14 |
| NT5C1B | 5’ nucleotidase, cytosolic 1B |
| MIR4757 | microRNA |
| OSR1 | Odd-skipped related transcription factor 1 |
| LINC00954 | Long non-coding RNA |
| TTC32 | Tetratricopeptide repeat protein 32 |
| WDR35 | WD repeat domain 35 |
| LOC101928222 | Long non-coding RNA |
| MATN3 | Matrilin 3 |
| LAPTM4A | Lysosomal protein transmembrane 4 alpha |
| SDC1 | Syndecan 1 |
| PUM2 | Pumilio RNA-binding family member 2 |
| RHOB | Ras homolog family member B |
| HS1BP3-IT1 | Intronic transcript, long non-coding RNA |
| HS1BP3 | HCLS1 binding protein 3 |
| GDF1 | Growth differentiation factor 7 |
| C2orf43 | mRNA |
| APOB | Apolipoprotein B |
| LOC645949 | Long non-coding RNA |
| LOC102723362 | Long non-coding RNA |
| KLHL29* | Kelch-like family member 29 |
| ATAD2B | ATPase family, AAA domain containing 2B |
| UBXN2A | UBX domain protein 2A |
| MFSD2B | Major facilitator superfamily domain containing 2B |

Note: the genes including and between the 2 genes marked with ‘*’ are within the 90% CI.
